# Generation and validation of novel adeno-associated viral vectors for the analysis of Ca^2+^ homeostasis in motor neurons

**DOI:** 10.1038/s41598-017-06919-0

**Published:** 2017-07-26

**Authors:** Rosa Pia Norante, Maria Lina Massimino, Paolo Lorenzon, Agnese De Mario, Caterina Peggion, Mattia Vicario, Mattia Albiero, Maria Catia Sorgato, Raffaele Lopreiato, Alessandro Bertoli

**Affiliations:** 1 Department of Biomedical Science, University of Padova, Padova, Italy; 2 CNR Neuroscience Institute, Padova, Italy; 3 Department of Medicine, and Venetian Institute of Molecular Medicine, Padova, Italy; 4 Present Address: Department of Integrative Medical Biology (IMB), Umeå Universitet, 901 87 Umeå, SE Sweden

## Abstract

A finely tuned Ca^2+^ homeostasis in restricted cell domains is of fundamental importance for neurons, where transient Ca^2+^ oscillations direct the proper coordination of electro-chemical signals and overall neuronal metabolism. Once such a precise regulation is unbalanced, however, neuronal functions and viability are severely compromised. Accordingly, disturbed Ca^2+^ metabolism has often been claimed as a major contributor to different neurodegenerative disorders, such as amyotrophic lateral sclerosis that is characterised by selective motor neuron (MN) damage. This notion highlights the need for probes for the specific and precise analysis of local Ca^2+^ dynamics in MNs. Here, we generated and functionally validated adeno-associated viral vectors for the expression of gene-encoded fluorescent Ca^2+^ indicators targeted to different cell domains, under the transcriptional control of a MN-specific promoter. We demonstrated that the probes are specifically expressed, and allow reliable local Ca^2+^ measurements, in MNs from murine primary spinal cord cultures, and can also be expressed in spinal cord MNs *in vivo*, upon systemic administration to newborn mice. Preliminary analyses using these novel vectors have shown larger cytosolic Ca^2+^ responses following stimulation of AMPA receptors in the cytosol of primary cultured MNs from a murine genetic model of ALS compared to the healthy counterpart.

## Introduction

Ca^2+^ ions play a fundamental role in all cell types by controlling an impressive number of signalling events. Therefore, a precise control of Ca^2+^ homeostasis in the cytosol and other cell compartments/organelles – performed by several systems like pumps, channels, transporters and Ca^2+^-buffering proteins – is essential for cell physiology. In neurons, local Ca^2+^ fluctuations have a key physiologic impact, by controlling the spatiotemporal pattern of electrochemical signals, neurite outgrowth, synaptic plasticity and the patterning of dendritic spines, which is of key importance in the neurochemical/neuroanatomical establishment of learning and memory, and cell survival. Not surprisingly, small changes in Ca^2+^ dynamics can lead to pathological conditions^1^, and derangement of Ca^2+^ homeostasis has been frequently indicated as a major contributor to the onset of various neurodegenerative disorders, such as Alzheimer’s, Parkinson’s, and Huntington’s disease^2^.

Among neurons, motor neurons (MNs) are particularly sensitive to noxious cell Ca^2+^ overloads because they possess low levels of Ca^2+^-buffering proteins^3, 4^, and express high levels of Ca^2+^-permeable α-amino-5-methyl-3- hydroxisoxazolone-4-propionate (AMPA) receptors that lack the GluR2 subunit. Such an architecture makes AMPA receptors more vulnerable to dysregulation of intracellular Ca^2+^ homeostasis and excitotoxicity^5^. Not surprisingly, perturbed Ca^2+^ homeostasis has been claimed as a major actor in the pathogenesis of different MN disorders, such as amyotrophic lateral sclerosis (ALS)^6, 7^ and spinal muscular atrophy^8^. The above notions highlight the need for molecular tools, such as suitable Ca^2+^ indicators, for the specific and precise analysis of Ca^2+^ movements in different sub-cellular compartments of MNs.

There are two major classes of Ca^2+^ indicators: chemical probes and gene-encoded calcium indicators (GECI). The first ones are hybridizations of small Ca^2+^-chelating molecules and a fluorophore. This class of probes include fura-2, quin-2, indo-1 and fluo-3, which were the first indicators used for Ca^2+^ measurements in cells^9^. A second class of probes is that of GECI, which includes different types of engineered proteins such as green fluorescent protein (GFP)-derived fluorescent probes, bioluminescent probes and fluorescence (or Förster) resonance energy transfer (FRET)-based indicators. A clear advantage of these probes is that – at difference from chemical indicators – they can be genetically targeted to different cell domains/compartments for local Ca^2+^ measurements. On the other hand, they suffer from the difficulty to be efficiently expressed in cells, in particular in primary neurons. Such a drawback, however, can be now easily overcome by use of viral expression vectors^10–12^.

Cameleon probes are based on FRET, a well-known quantum mechanical process consisting in the energy transfer from an exited fluorescent donor to an acceptor fluorophore placed in close proximity. Therefore, when FRET occurs, an increased fluorescence of the acceptor, together with a decreased fluorescence of the donor can be observed and quantified. In cameleons, the FRET donor and acceptor are GFP-derived fluorescent proteins separated by a Ca^2+^-sensing domain, consisting of calmodulin (CaM) and a CaM-binding peptide derived from the myosin light chain kinase M13. Upon Ca^2+^ binding to this domain, the FRET donor and acceptor are brought in close spatial proximity, thereby allowing FRET to occur. To date, the most used GFP-modified proteins in cameleons are the yellow fluorescent protein (YFP, acting as donor) and the cyan fluorescent protein (CFP, acceptor). In last years, several studies based on site-directed mutagenesis have led to the generation of improved cameleon Ca^2+^ probes, by increasing the brightness of the FRET acceptor in response to Ca^2+^ fluctuations, reducing the possible competitive effect of endogenous CaM, and extending the affinity of cameleons for Ca^2+^ to render the probes suitable for Ca^2+^ measurements in different cell districts. In particular, different kinds of cameleon exist (classified as D1, D2, D3 and D4^13^) covering a wide K_d_ range for Ca^2+^ that allow scientists to choose the best probe for the specific cell compartment under study^13–15^.

In this work, we have generated and functionally validated adeno-associated viral (AAV)-based vectors for the expression of cameleons targeted to different cell domains [i.e., cytosol, mitochondrial matrix, or endoplasmic reticulum (ER) lumen] under the transcriptional control of a MN-specific promoter. We demonstrate that such probes are specifically expressed in MNs (and not in other cell types) in primary cell cultures from the mouse spinal cord, and that they allow reliable Ca^2+^ measurements in the target cell compartment. Pilot experiments also demonstrate altered Ca^2+^ homeostasis in the cytosol of primary MNs from a genetic model of ALS, and that the generated AAV expression vectors can be suited for the *in vivo* expression of cameleons in spinal cord MNs in mice.

## Results

Given the plausible implication of perturbed Ca^2+^ homeostasis in the pathogenesis of MN diseases^6–8^, in this study we sought to generate and validate expression systems for GECI that were suited for the assessment of local Ca^2+^ fluctuations in MNs. To this purpose, we have engineered AAV plasmids (pAAV) for the expression of cameleon probes targeted to the cytosol (pAAV-[Hb9_AB]-D1cpv), the mitochondrial matrix (pAAV-[Hb9_AB]-4mtD3cpv) and the ER lumen (pAAV-[Hb9_AB]-D4ER), under the control of a MN-specific, homeobox Hb9-derived, promoter (Supplementary Fig. S1). The cameleon probes of choice have great ratiometric sensitivity and large dynamic range, thereby allowing to detect small changes in Ca^2+^ concentration over the noise in the target compartment^13^.

**Figure 1:**
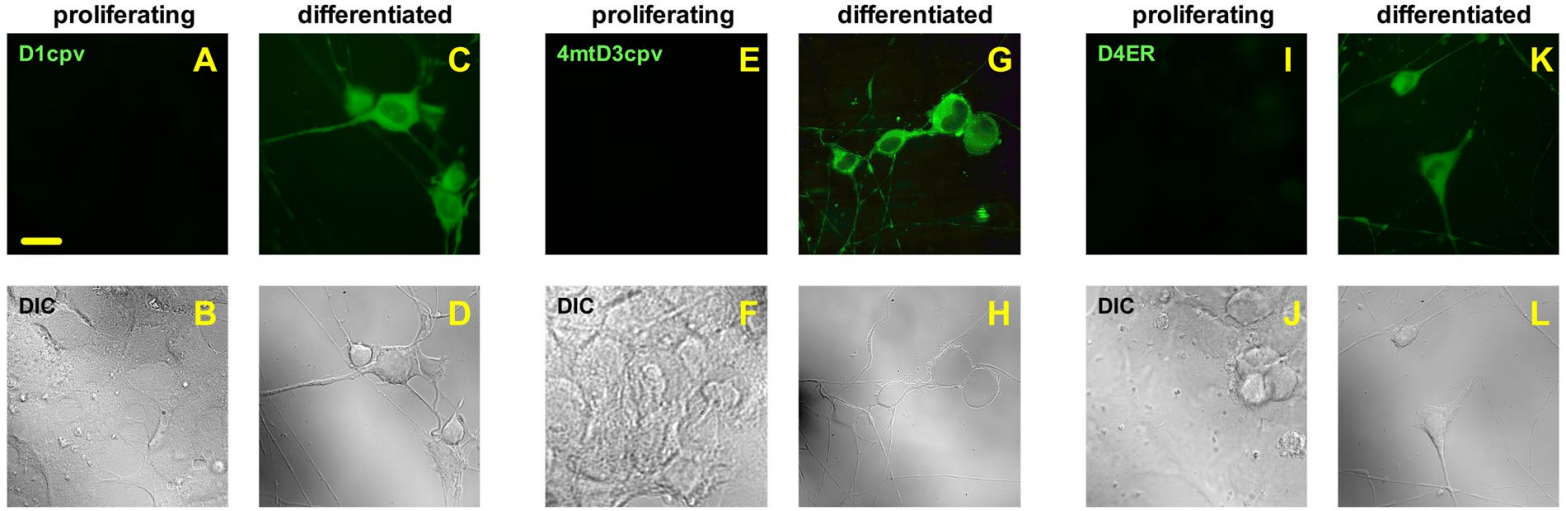
The cameleon probes under the control of the Hb9-derived promoter are expressed in differentiated, but not in proliferating, NSC-34 MN cells. NSC-34 cells were infected with the AAV vectors coding for the Hb9_AB-driven, MN-specific, cameleon probes targeted to the cytosol (D1cpv, panels A–D), the mitochondrial matrix (4mtD3cpv, panels E–H), or the ER lumen (D4ER, panels I–L), and cultured under non-differentiating (proliferating, panels A,B,E,F,I,J) or differentiating (by growth in the presence of retinoic acid 5 μM, 192 h, panels C,D,G,H,K,L) conditions. Fluorescence (λ_ex_ = 488 nm, λ_em_ = 526/550 nm) and differential interference contrast (DIC) micrographs of representative fields were taken with a suited microscope equipped with a CCD camera. No fluorescent cell was observed in non-differentiated NSC-34 cultures, while the fluorescent Ca^2+^ probes were expressed in cells differentiated towards a MN phenotype. Shown data are representative of at least 3 independent experiments yielding comparable results. Scale bar = 20 µm.

To validate such vectors for the specific recording of Ca^2+^ fluxes in MNs, we firstly analysed the expression of our AAV-driven probes in the immortalised NSC-34 cell line that – when properly differentiated – displays several typical properties of MNs^16, 17^, including the transcriptional activation of the Hb9 gene^18^. We therefore checked the expression of the three cameleon probes in NSC-34 cells, transduced with the AAV vectors, either cultured under proliferating conditions or induced to differentiate by treatment with retinoic acid. Under the latter culturing conditions, all cells were successfully differentiated into a MN phenotype, as determined by both morphological observations (Fig. 1, bright-field images of panels D,H,L) and immunoblot assessment of the MN marker choline acetyl-transferase (Supplementary Fig. S2). We observed that all cameleons were abundantly present in differentiated cells (>97% cells expressing the probes), but completely absent in cells under active proliferation, suggesting that the Ca^2+^ probes were specifically expressed in cells resembling a MN phenotype (Fig. 1).

**Figure 2:**
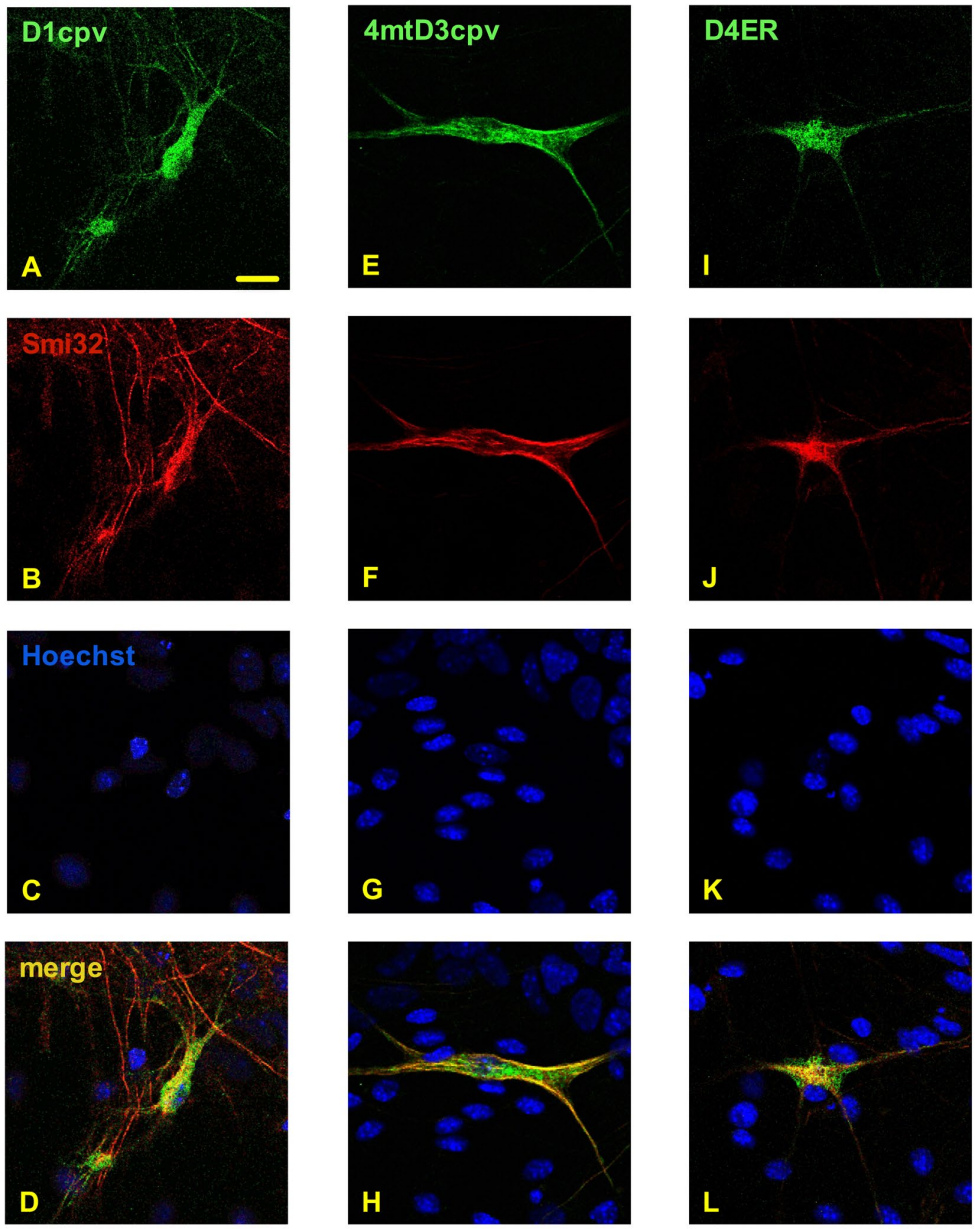
The cameleon probes are selectively expressed in motor neurons in mouse spinal cord primary cultures. Primary cell cultures from the mouse spinal cord were transduced with the AAV vectors coding for the cameleons targeted to the cytosol (panels A–D), the mitochondrial matrix (panels E–H) or the ER lumen (panels I–L). After 12 days of culturing, cells were fixed, permeabilised, immunostained with an antibody to the motor neuron (MN) marker SMI32, and then counter-stained with the nuclear fluoro-probe Hoechst 33342. Confocal microscope images of cells were then collected after excitation at either λ = 488 nm for visualising the fluorescent Ca^2+^ probes (green signal, panels A,E,I), λ = 543 nm for visualising SMI32-positive cells (red signal, panels B,F,J), or λ = 405 nm for visualising the nuclei of all cells (blue signal, panels C,G,K). Merging of the three fluorescent channels (panels D,H,L) shows that only SMI32-positive MNs express the Ca^2+^ probes (see also Supplementary Fig. S3). Scale bar = 20 µm.

We then analysed by confocal microscopy the expression of cameleons in primary cultures from mouse spinal cord. After 12 days of growth, such cultures contained different cell types, including mature MNs resembling those present *in vivo*, both morphologically and for the expression of specific molecular markers, such as the neurofilament protein SMI32, with 4.9% ± 0.7% of SMI32-positive MNs over total cells (n = 35). As described in Fig. 2, after transduction of such cell cultures with the AAV vectors, the cameleons (green signal, panels A,E,I) were present in SMI32-positive cells (red signal, panels B,F,J), while in no case other cell types (highlighted by nuclear staining, blue signal, panels C,G,K) were positive for the expression of cameleons, providing evidence that the Hb9 promoter-driven expression of the Ca^2+^ probes is specific for MNs (merge, panels D,H,L). In addition, the transduction efficiency of MNs was rather high, given that in all cases more than 70% of SMI32-positive cells were also positive for the expression of the cameleon probes (Supplementary Fig. S3).

The MN-specific expression of the Ca^2+^ probes was further reinforced by the finding that the AAV vectors were unable to drive the expression of the mitochondria-targeted cameleon in primary cultures of cortical, hippocampal or cerebellar granule neurons, or spinal astrocytes (Supplementary Fig. S4).

**Figure 3:**
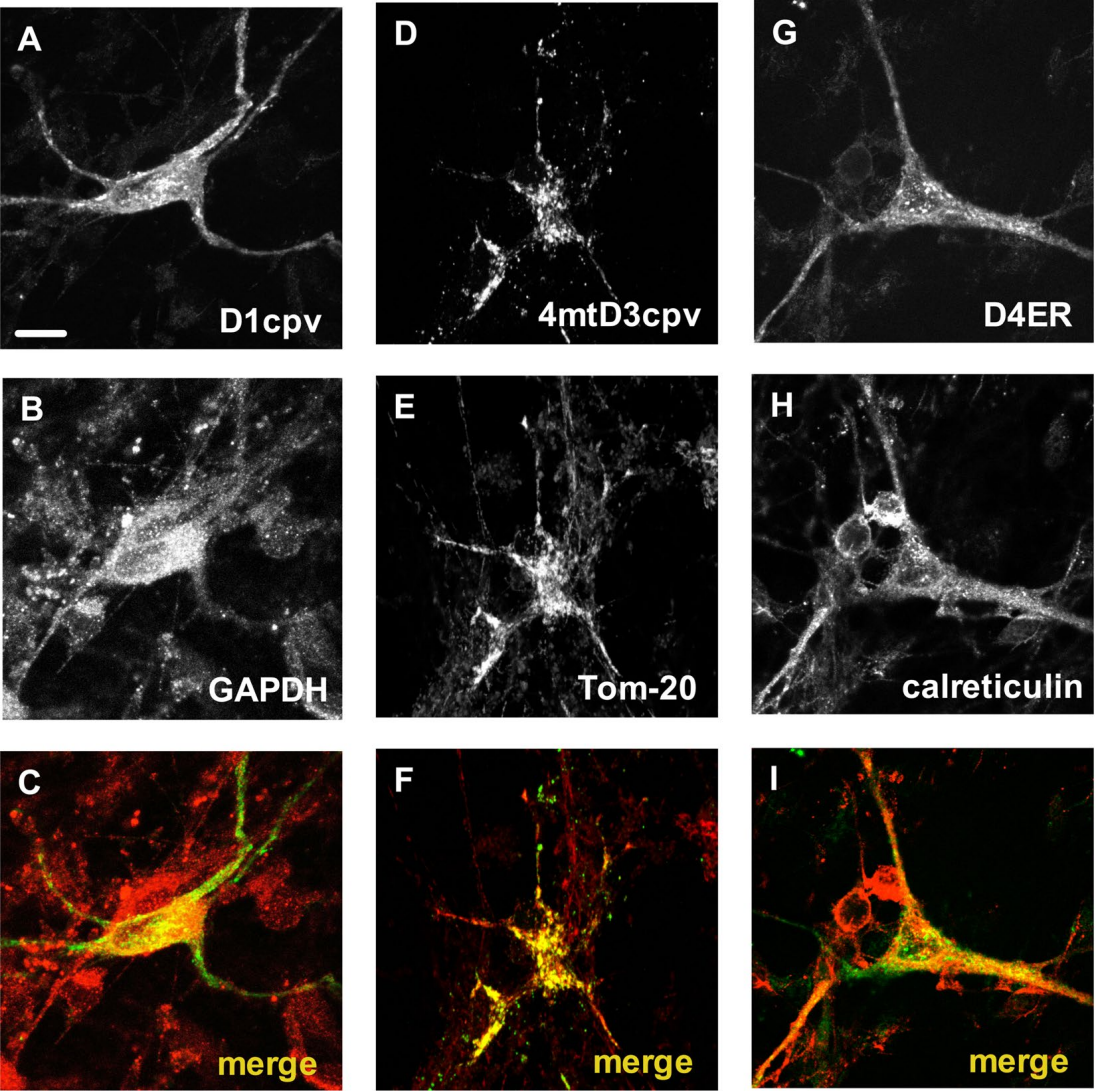
The cameleon probes correctly localize to the target sub-cellular compartment in primary MNs. Primary cell cultures from the mouse spinal cord were transduced with the AAV vectors coding for the cameleons targeted to the cytosol (panels A–C), the mitochondrial matrix (panels D–F) or the ER lumen (panels G–I). After 12 days of culturing, cells were fixed, permeabilised, and immunostained with antibodies to proteins present in the cytosol (GAPDH, panel B), mitochondria (Tom-20, panel E), or the ER (calreticulin, panel H). Images report the merged reconstruction of complete z-stacks of cells, collected by a confocal microscope after excitation at either λ = 488 nm for visualising the fluorescent Ca^2+^ probes (panels A,D,G), or λ = 543 nm for visualising the immuno-labeled proteins (panels B,E,H). The remarkable fluorescence overlapping of each targeted cameleon and the corresponding marker immunosignal (panels C,F,I) demonstrates the correct sub-cellular targeting of all used Ca^2+^ probes. Scale bar = 10 µm.

Although the correct sub-cellular targeting of the used cameleon chimeric constructs has been already demonstrated in different cell paradigms^14, 19, 20^, we checked that the probes had the expected localisation also in primary MNs. As shown by the confocal microscopy z-stack reconstructions of Fig. 3, each cameleon (panels A,D,G) largely co-localised (yellow signal, merged images of panels C,F,I) with immuno-labelled markers of the corresponding target compartment, i.e., glyceraldehyde 3-phosphate dehydrogenase (GAPDH) for the cytosol (panel B), Tom20 for mitochondria (panel E), and calreticulin for the ER lumen (panel H), indicating that the probes are correctly processed and delivered to the target site in AAV transduced MNs.

ALS is a fatal neurodegenerative disorder that occur on sporadic or genetic grounds, leading to a selective and progressive loss of MNs in the spinal cord, brainstem and cerebral cortex, thereby resulting in severe motor deficits and – eventually – to death^21, 22^. Since perturbations of Ca^2+^ homeostasis in MNs have been often claimed as a crucial event in disease progression^7, 23^ we tested the applicability of the novel Ca^2+^-probe encoding vectors to the comparative analysis of local Ca^2+^ homeostasis in MNs from an ALS mouse model [i.e., mice expressing the ALS-related G93A mutant of human (h) SOD1 and the healthy counterpart (hSOD1(WT)-expressing mice)].

**Figure 4:**
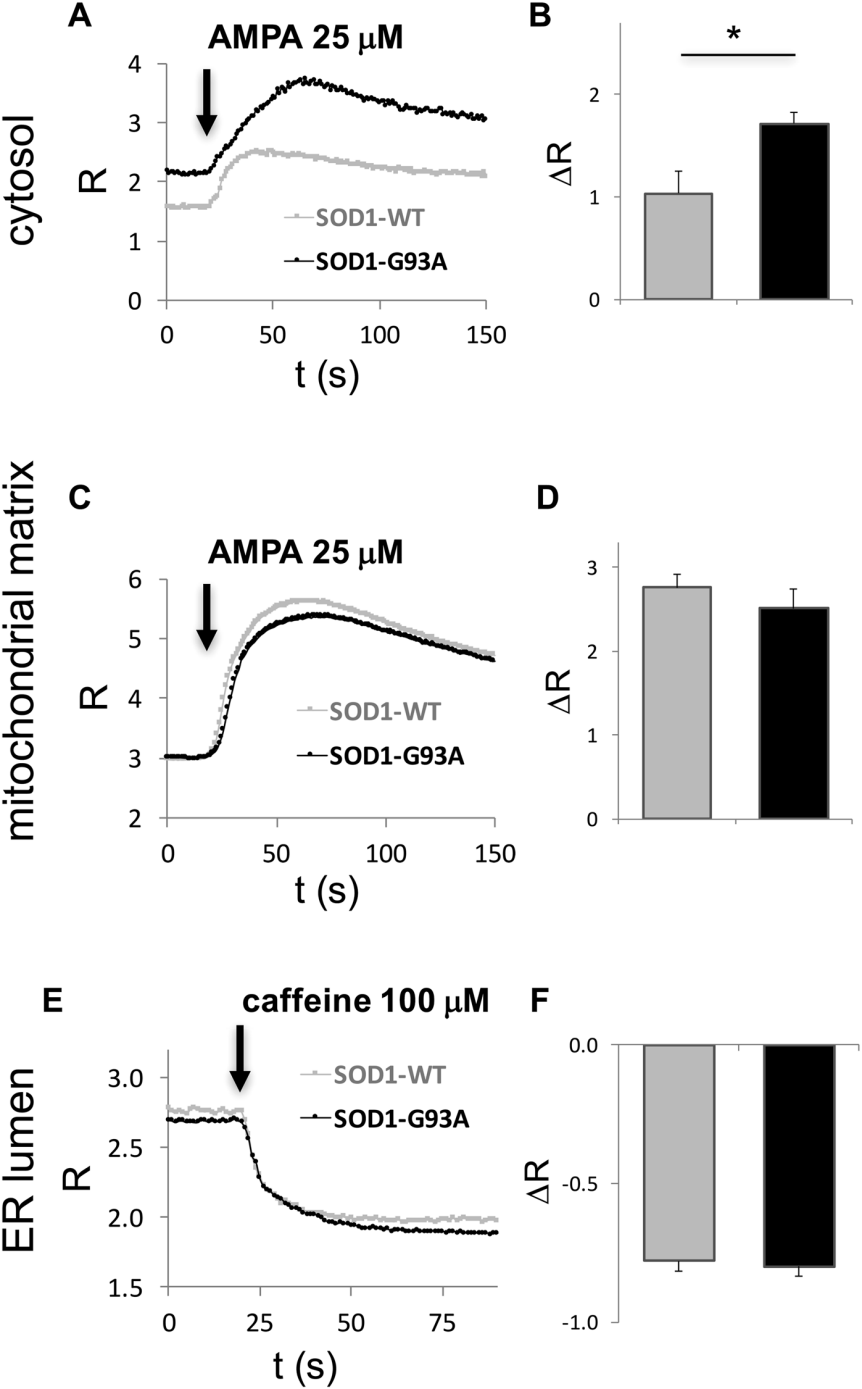
AMPA stimulation results in higher cytosolic Ca^2+^ transients in primary spinal cord MNs from ALS mice than in the healthy counterpart. Primary spinal cord cultures from hSOD1(WT) or hSOD1(G93A) mice were transduced with the AAV vectors coding for the cameleon probes targeted to the cytosol (panels A,B), the mitochondrial matrix (panels C,D) or the ER lumen (panels E,F). After 12 days of culturing, FRET measurements were performed on single MNs expressing the different Ca^2+^ probes using a computer-assisted fluorescence microscope equipped with a suitable system for double-wavelength recordings. The ratio (R) between the FRET acceptor and donor was calculated by the data acquisition software, allowing the comparison of Ca^2+^ mobilisation following stimulation with AMPA (25 μM in the presence of 2 mM CaCl_2_, for the cytosol and the mitochondrial matrix) or caffeine (100 μM, for the ER) at the time-points indicated by arrows, between hSOD1(WT) or hSOD1(G93A) MNs. Panels A, C, E report average traces of the Ca^2+^ dynamics (for the sake of clarity, error bars are not reported), while bar diagrams of panels B,D,F report the mean difference between FRET ratios (ΔR = R_peak_ − R_baseline_) following the indicated stimulus in the three cell compartments. While no difference was recorded in Ca^2+^ movements in the mitochondrial matrix or the ER lumen, the cameleon-based approach highlighted a significantly higher Ca^2+^ response following AMPA stimulation in the cytosol of MNs expressing the ALS-related hSOD1(G93A) mutant compared to the hSOD1(WT) counterpart. Data are reported as mean ± standard error of the mean (SEM); n = 6 (A and B), 12 (C and D), 9 (E and F), for each genotype, from at least 3 different MN cultures for each condition; *p < 0.01, Student’s t-test.

In this work we did not calculated absolute Ca^2+^ concentrations, which, however, could be determined by suitable calibration procedures^13^. Instead, we simply reported the ratio between the acceptor and the donor fluorescence of the FRET-based probes, which is, nevertheless, sufficient for comparative analyses. Such experiments demonstrated that all cameleon probes were functional, and indicated that hSOD1(G93A)-expressing MNs have larger cytosolic Ca^2+^ transients following AMPA stimulation, compared to SOD1(WT) MNs (Fig. 4A,B). Comparable results for both basal and peak Ca^2+^ levels were obtained using the chemical Ca^2+^ indicator Fura-2 (Supplementary Fig. S5), further supporting the suitability of the AAV-mediated, Hb9_AB-driven, expression of the cameleon probes for reliable Ca^2+^ measurements in MNs. Conversely, the mitochondrial targeted cameleon revealed no significant difference in AMPA-stimulated Ca^2+^ fluxes in the mitochondrial matrix (Fig. 4C,D). Also the ER-targeted cameleon was properly functioning, allowing the measurement of ER Ca^2+^ discharge upon stimulation with caffeine, which, however, evidenced no difference between MNs with the two genotypes (Fig. 4E,F).

**Figure 5:**
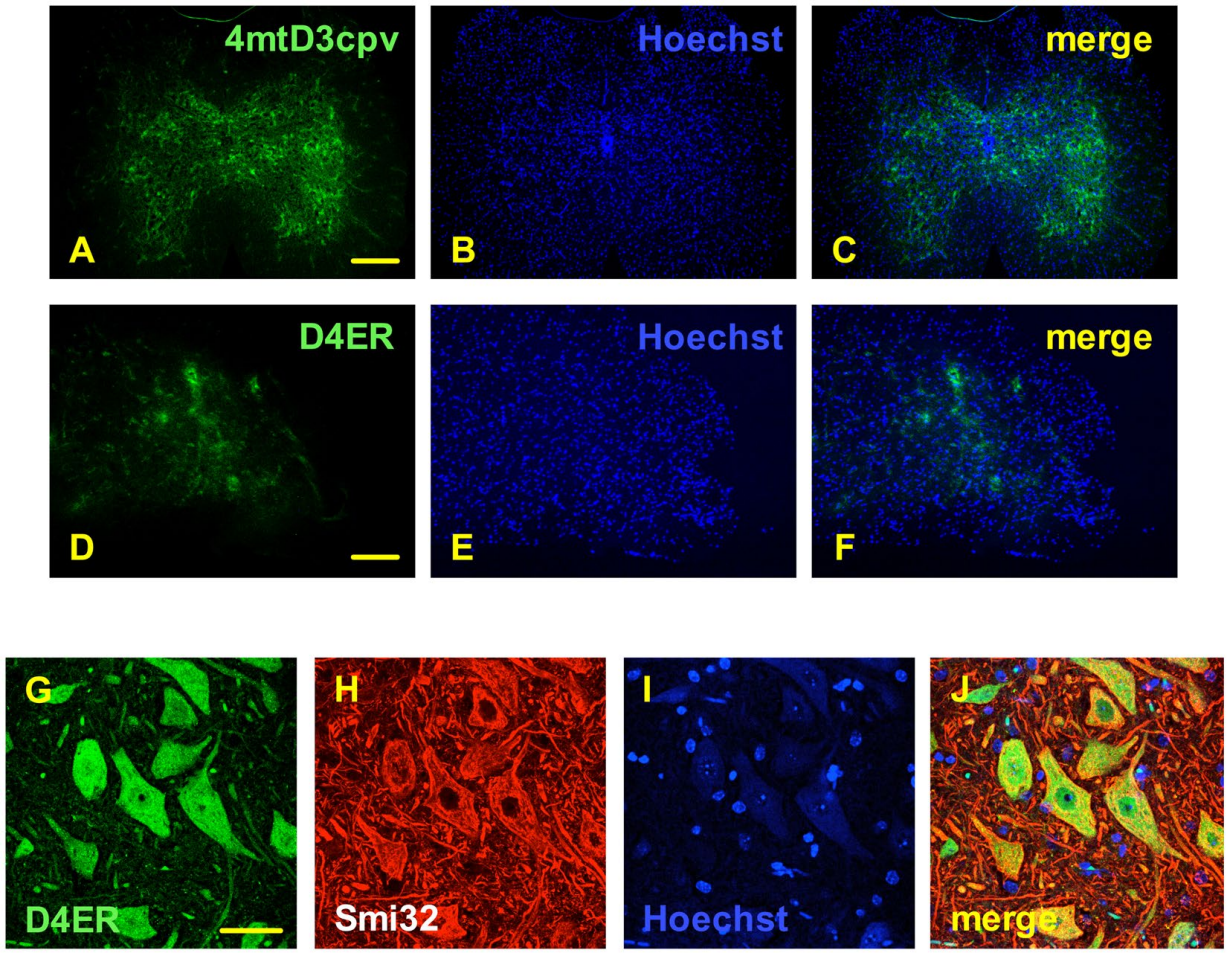
The MN promoter-driven cameleons can be efficiently and specifically expressed in MNs after transduction of mice with the AAV vectors *in vivo*. Neonatal mice (P1) were injected into the temporal vein with AAV vectors encoding the mitochondrial (panels A–C) or the ER (panels D–J) cameleon. Mice were sacrificed 4 weeks after injection, and spinal cord cross-sections were labeled with the nuclear fluorescent dye Hoechst 33342 (panels B,E,I) and immunostained with the MN marker SMI32 (panel H), and then observed with a fluorescence stereo-microscope (panels A–F) or a confocal microscope (panels G–J). Low-resolution stereo-microscopy images show a diffuse expression of both fluorescent Ca^2+^ probes (green signal, panels A,D,G), indicating that the cameleons are effectively transduced and expressed in the spinal cord *in vivo*. The merged reconstruction of complete z-stacks of cells collected by confocal microscopy shows that the D4ER cameleon is expressed only in SMI32-immunopositive MNs (merged image, panel J), suggesting the cell-specific expression of the probe. Scale bars = 200 µm (A–C), 100 µm (D–F), 10 µm (G–J).

Finally, we probed if the developed AAV-based vectors were suited for the *in vivo* expression of the cameleon probes. To this purpose, we injected the AAV vector coding for the mitochondrial or the ER cameleon into the superficial temporal vein of newborn mice, and (4 weeks later) we evaluated the expression of the probes in spinal cord sections. By use of a fluorescence stereo-microscope, we observed an intense and diffuse signal in tissue samples of mice transduced with either the mitochondrial (Fig. 5A–C) or the ER (Fig. 5D–F) cameleon, providing a transduction ratio (cameleon-positive cells over total cells) of 16% ± 4% and 9.8% ± 1.7%, respectively (n = 3). That the AAV-based expression system of cameleons was specific for MNs also *in vivo* was demonstrated by the immunostaining of the MN marker SMI32 in spinal cord slices from ER cameleon-infected mice, followed by confocal microscopy (Fig. 5G–J).

## Discussion

Recombinant AAVs are a powerful means for *in vivo* gene delivery^24, 25^ and for the transduction of primary cell cultures that are particularly hard to transfect, such as neurons^26, 27^. AAVs display low cytotoxicity and immunogenicity, are safe for the operator, and are suitable for long term gene expression in non-dividing cells with relatively high gene delivery efficiency, depending on the cell type^28^.

In this work, we have generated pAAV plasmids encoding cameleon Ca^2+^ probes, genetically targeted to different cell compartments, under the transcriptional control of a MN-specific promoter. Given that AAV vectors has limited cloning capacity (i.e., large plasmids can hardly be packaged into AAV viral particles), we have chosen a genetically engineered minimal promoter of the Hb9 gene (Hb9_AB), which is about 550 bp in length and has already been demonstrated to specifically drive the expression of a transgene into MNs, both *in vitro* and *in vivo* ^29^. For the preparation of the viral particles, we have chosen the AAV9 serotype because of its broad tropism, including the capacity to target neurons^28^. We have demonstrated that the engineered AAV vectors are suitable for the specific expression of the cameleons targeted to the cytosol, the mitochondrial matrix and the ER lumen of MNs from spinal cord primary cultures of mice, and allow measuring Ca^2+^ responses to proper stimuli in such cellular districts. These vectors have several advantages with respect to other expression systems or the use of ubiquitous promoters. Indeed, they allow Ca^2+^ measurements in single primary MNs with no need for difficult and time-consuming MN purification steps. In addition, MN Ca^2+^ measurements can be performed in a more physiologic environment, containing glial cells and other neuronal types, compared to pure MN cultures.

Although SMI32-positive MNs were less than 5% of total cells in our primary spinal cord cultures, the transduction efficiency ( >70%) of MNs by the AAV-mediated infection system was largely sufficient for single cell analyses. Indeed, the transduction rate was much higher than that achieved by transfection with plasmidic vectors, with which cameleon-expressing MNs were rarely found in each preparation, also when using a constitutive and ubiquitous (cytomegalovirus), or the pan-neuronal human synapsin1 (hSyn), promoter (data not shown). In addition, in our experimental setting, a substantial number of SMI32-positive cells contained the Ca^2+^ probes at an expression level that easily allowed single cell recordings of Ca^2+^ dynamics.

Among GECI, the ratiometric cameleon probes have several advantages, including low risk of artifacts, high sensitivity, good dynamic range, and the possibility to provide absolute Ca^2+^ concentration values upon proper calibration procedures^13^. The ratiometric nature of these Ca^2+^ indicators, as in the case of chemical probes such as Fura-2, prevents any differences due to level of expression or cell thickness. In addition, engineering of mutant cameleons have provided researcher with several cameleons displaying a wide range of K_d_ for Ca^2+^, thereby allowing reliable measurements of basal Ca^2+^ levels and evoked Ca^2+^ movements depending on the target cell compartment, the cell type and the desired stimulation protocol^14, 19^.

For cytosolic Ca^2+^ measurements we have used the archetypal D1-based probe^30^, which allowed us to measure cytosolic fluctuations of the ion in MNs following AMPA stimulation.

Mitochondria-Ca^2+^ interplay is essential for cell physiology. On the one hand, the fine regulation of mitochondrial Ca^2+^ controls ATP production, because different mitochondrial enzymes rely on local Ca^2+^ concentration for their activity. On the other hand, mitochondria are a major Ca^2+^-buffering system avoiding noxious cytosolic Ca^2+^ overloads. While it is assumed that resting mitochondrial Ca^2+^ concentration is in the order of 10^−1^ μM, upon stimulation promoting Ca^2+^ entry from the extracellular space or its release from the ER, Ca^2+^ levels in the mitochondrial matrix may reach up to 10^2^ μM, depending on the cell type and the stimulus^31^. Therefore, we have selected the 4mtD3cpv cameleon that, for its K_d_ and dynamic range, has been proved useful for measuring mitochondrial Ca^2+^ dynamics^13^.

Finally, for the measurements of Ca^2+^ homeostasis in the ER lumen, we have chosen the recently generated D4ER probe^14^ that has been proved useful for comparative Ca^2+^ measurements in an experimental setting of neurodegenerative disorders^32^.

Notably, cameleons are mostly expressed in SMI32-positive α-MNs, enriched in the anterior ventral horn of the spinal cord^33, 34^, which are particularly vulnerable to (AMPA-mediated) excitotoxic challenge and ALS-related damage^6, 7^. Thus, to prove the efficacy of the probes to monitor local Ca^2+^ fluctuations in MNs, we have compared the response of MNs from mice expressing the ALS-related hSOD1(G93A) mutant, or the hSOD(WT)-expressing counterpart, to selected stimuli. In particular, because MNs express high levels of AMPA receptors through which they process strong glutamatergic inputs, and because excitoxicity has been proposed to contribute crucially to MN injury in ALS, probably as a consequence of glutamate-triggered Ca^2+^ overload^7, 35^, we investigated Ca^2+^ responses to AMPA stimulation. The cytosolic and mitochondrial probes allowed the assessment of AMPA-mediated cell Ca^2+^ responses, thereby providing proof-of-principle for the functional application of these tools. Such conclusion is further corroborated by the finding that Fura-2-based measurements provided very similar results for cytosolic Ca^2+^ responses to AMPA stimulation. In addition, both cameleon- and Fura-2-based strategies underscored a larger cytosolic Ca^2+^ response in ALS MNs compared to the healthy controls supporting the idea that Ca^2+^ overloads may be involved in ALS MN damage^7, 36^. Surprisingly, in spite of increased cytosolic Ca^2+^ load, no difference was observed in the mitochondrial matrix, suggesting a possible deficit of mitochondrial Ca^2+^ upload in ALS MNs that deserves further investigation. With this respect, it must be noted that, to the best of our knowledge, no direct measuring of mitochondrial Ca^2+^ fluctuations under our experimental settings has been previously reported, and that mitochondrial Ca^2+^ handling deficits could be stage-dependent during ALS disease course^37^. We also provided evidence that the ER cameleon was properly functioning and monitored the ER Ca^2+^ discharge promoted by caffeine^38^, although no difference was observed between the two SOD1 genotypes. These notions further highlight the importance of the here-presented tools for deepening our understanding of disease-associated local Ca^2+^ alterations in MNs, in *in vitro* and *in vivo* models.

In conclusion, the novel vectors we generated in this work allowed for the first time to directly monitoring local Ca^2+^ fluctuations in sub-cellular compartments of primary mouse MNs, thereby providing valuable and versatile tools for expanding our knowledge on Ca^2+^-related pathogenic routes in ALS and other neuromuscular diseases. With this respect, it is worth underlining that other GECI-encoding sequences can be easily cloned into the generated MN promoter-containing pAAV scaffold, thus further increasing the toolset of Ca^2+^ probes with different properties (e.g., K_d_) for suitable Ca^2+^ measurements under different experimental protocols in MNs.

Importantly, we also provided evidence that the AAV vectors can drive the selective *in vivo* expression of the Ca^2+^ probes in MNs. This result was achieved by systemic administration of the virus, which is by far less invasive and arduous than local injection in the spinal cord of live mice. Thus, by means of suitable fluorescence microscopy equipment (e.g., two-photon microscopy), this system could be used for Ca^2+^ measurements in more physiologic environments than primary cultures, such as tissue slices or even live animals.

## Materials and Methods

### Plasmid construction

Multiple pAAVs have been generated, for the expression of the FRET-based cameleon Ca^2+^ probes targeted to different cellular compartment (i.e. cytosol, mitochondrial matrix and ER lumen^13, 14, 19, 20^ under the control of either a minimal promoter (Hb9_AB) derived from the (MN)-specific promoter of the Hb9 gene^29^, or the pan-neuronal hSyn promoter.

Firstly, the sequence coding for the cytosolic probe (D1cpv)^19^, previously isolated by BamHI-EcoRI cleavage of a pcDNA3-D1cpv plasmid (kindly provided by Dr. Roger Tsien, University of California, San Diego, USA)^13, 19^, was inserted into the BamHI-EcoRI sites of the pAAV-[hSyn]-ChR2/EYFP vector (Addgene, cat. # 26973) (Supplementary Fig. S1, panel A) by T4 DNA ligase, thus providing the pAAV-[hSyn]-D1cpv plasmid (Supplementary Fig. S1, panel B).

For the generation of the ER lumen-targeted expression vector, the sequence coding for the D4ER cameleon^14^ was amplified by PCR using the pcDNA3-D4ER plasmid (kindly provided by Dr. Paola Pizzo, Dept. of Biomedical Science, Univ. of Padova) as template, and the primers ER-KpnI-F and ER-HindIII-R (sequences are reported in the Supplementary Information). The PCR product was digested with KpnI-HindIII, and purified DNA fragments were ligated into the KpnI-HindIII sites of the pAAV-[hSyn]-ChR2/EYFP as indicated above, generating the pAAV-[hSyn]-D4ER plasmid (Supplementary Fig. S1, panel B).

Subsequently, the [hSyn] promoter in the obtained plasmids was substituted with the sequence coding for the ∼550 bp MN specific promoter [Hb9_AB], which was previously amplified by PCR using the Bg.Hb9_AB_GFP plasmid^29^ (kindly provided by Dr. Caterina Bendotti, IRCCS Istituto di Ricerche Farmacologiche Mario Negri, Milano, Italy) as template, and primers AB-MluI-F and AB-KpnI-R (sequences are reported in the Supplementary Information). The PCR product, subjected to MluI-KpnI cleavage and purified, was then ligated into both pAAV-[hSyn]-D1cpv and pAAV-[hSyn]-D4ERcpv plasmidic fragments digested with the same enzymes.

Concurrently, the pAAV vector encoding the mitochondrial matrix-targeted 4mtD3cpv^13^ cameleon was obtained by replacing the [hSyn] promoter sequence of the pAAV-[hSyn]-4mtD3cpv plasmid (a generous gift by Dr. Paola Pizzo, Dept. of Biomedical Science, Univ. of Padova) with the [Hb9_AB] sequence, by using the MluI and KpnI restriction sites.

After transformation of *E. coli* TOP10 cells with the generated constructs and selection of positive clones, recombinant plasmids were amplified and validated by sequencing. All enzymes were from New England Biolabs, excepted the high-fidelity DNA polymerase (KAPA Biosystems), and DNA primers (Life Technologies). DNA manipulations have been performed accordingly to standard methods^39^ and to manufacturer’s instructions. Detailed maps and plasmids sequences are all available upon request to the corresponding author.

AAV viral particles (AAV9 serotype) were produced by Vigene Biosciences, providing viral titers ranging from 2 × 10^13^ to 10 × 10^13^ GC/ml (see Supplementary Information).

### Animals

Tg mice expressing WT, or the ALS-related G93A mutant of hSOD1, (B6SJL(Tg-SOD1)2Gur/J and B6SJL(Tg-SOD1*G93A)1Gur/J mice, respectively) were purchased from The Jackson Laboratories. The colonies were maintained by breeding hemizygote (Tg) males to wild-type B6SJLF1/J hybrid females. Embryos and newborns were genotyped (as described in Supplementary Information), and used for subsequent experiments. All aspects of animal care and experimentation were performed in compliance with European and Italian (D.L. 26/2014) laws concerning the care and use of laboratory animals. All experimental procedures and animal care protocols were approved by the Italian Ministry of Health (authorization N. 305/2017-PR), and by the Ethical Committee for animal care and use of the University of Padova (*OPBA*). All efforts were made to minimize animal suffering and reduce the number of animals used in the experiments.

### Primary cultures

Primary MN cultures were established from E12.5 mouse embryos according to the Henderson’s protocol (Mettling *et al*.)^40^. Briefly, spinal cords were dissected from individual embryos in Hybernate medium (Gibco) added with 2% (v/v) B27 supplement (Gibco), and tissue was cut (<1 mm pieces) and incubated (8 min, 37 °C) in a buffer containing NaCl (124 mM), KCl (5.4 mM), NaH_2_PO_4_ (1 mM), glucose (3.6 mM), HEPES (25 mM, pH 7.4), added with BSA [0.3% (w/v)] and trypsin [0.025% (w/v)].

Then, cells were gently dissociated in a buffer made of Leibovitz’s medium (L15, Gibco) supplemented with sodium bicarbonate [7.5% (w/v)], horse serum [HS, 2% (v/v)], glucose [7.2% (w/v)], progesterone [0.1% (w/v)], insulin [1% (w/v)], putrescine [1% (w/v)], conalbumin [1% (w/v)], sodium selenite [0.1% (w/v)], BSA [0.4% (w/v)] and DNase I [0.1 mg/ml].

The cell suspension was then centrifuged (500 × g, 5 min, RT) over a cushion of BSA [4% (w/v)] (in the above medium), after which cells were recovered from the bottom of the tube, resuspended in a MN culture medium consisting of Neurobasal medium (Gibco) supplemented with HS [2% (v/v)], B27 [2% (v/v)], L-glutamine (0.05 mM), glutamate (25 µM), mercaptoethanol (25 µM), BDNF (10 ng/ml), GDNF (10 ng/ml), CNTF (10 ng/ml), penicillin (100 U/ml), and streptomycin (100 µg/ml). Cells were then plated at a density of approximately 500,000 cells/well in 12-wells culture plates containing (18 mm diameter) glass coverslips coated with poly-D-ornithine (1.5 µg/ml in sterilized bidistilled H_2_O, 2 h, RT) and then with laminin (3 µg/ml in L15 medium, 3 h, 37 °C in a 5% CO_2_ atmosphere), and grown (37 °C in a 5% CO_2_ atmosphere).

Primary cultures of cerebellar granule and cortical neurons were prepared as described in De Mario *et al*.^41^. Hippocampal neurons were prepared following the same procedures for cortical neurons, except that they were grown in Neurobasal medium added with foetal bovine serum [FBS, 10% (v/v)].

Primary cultures of spinal astrocytes were isolated from newborn mice and cultured as described previously in Martorana *et al*.^42^. Once the cultures reached the confluence, they were re-plated at the optimal density 80,000 cells/well in 24-well plates containing glass coverslips and maintained in minimal essential medium (MEM, Gibco), supplemented with FBS [10% (v/v)], L-glutamine (2 mM), glucose [0.3% (w/v)] in phosphate buffered saline (PBS), penicillin (100 U/ml]), and streptomycin (100 µg/ml).

On day 2 from plating, all primary cultures were transduced with the AAV particles and used in experiments at different times depending on the cell type. For the transfection of cortical neurons and spinal astrocytes with plasmidic vectors, the Lipofectamine-2000 reagent (Gibco) was used, following the manufacturer’s instructions.

### Immunocytochemistry

For immunocytochemical analysis, cells were firstly washed in ice-cold PBS, and fixed (20 min, RT) in paraformaldehyde [PFA, 4% (w/v)] in PBS. After washing in PBS, cells were permeabilized in PBS containing Triton X-100 [1 h, RT, 0.02% (w/v)] and then incubated (overnight, 4 °C) with the following primary antibodies [Ab, diluted in PBS containing 1% (w/v) BSA as indicated in parentheses]: anti-SMI32 mouse monoclonal (m) Ab (1:200, Covance, cat. n. SMI-32R & SMI-32P); anti-glial fibrillary acidic protein (GFAP) rabbit polyclonal (p) Ab (1:500, Dako, cat. n. Z0334); anti-microtubule-associated protein-2 (MAP-2) chicken pAb (1:1000, Abcam, cat. n. ab5392); anti-calreticulin rabbit pAb (1:50, Stressgen, cat. n. ADI-SPA-600-J); anti-GAPDH rabbit pAb (1:50, Santa Cruz Biotechnologies, cat. n. sc-25778); anti-Tom20 rabbit pAb (1:50, Santa Cruz Biotechnologies, cat. n. sc-11415). After extensive washings in PBS, cells were incubated (1 h, 37 °C) with the following secondary antibodies, depending on the used primary Ab: Alexa Fluor 555-conjugated anti-mouse IgG (1:500, Molecular Probes); rhodamine-conjugated (1:100, Dako) or Alexa Fluor 568-conjugated (1:200, Molecular Probes) anti-rabbit IgG; Alexa Fluor 568-conjugated anti-chicken IgG (1:500, Molecular Probes). Cell nuclei were counter-stained with Hoechst 33342 (5 μg/ml, Sigma), and coverslips were finally washed in PBS, mounted in montage solution [8% Mowiol 40–88 (Sigma) in glycerol and PBS (1:3 (v/v)], and observed with an inverted fluorescence microscope (Leica CTR6000) equipped with a computer-assisted charge-coupled camera (Orca Flash 4.0, Hamamatsu), or with a Leica TSC SP5 inverted confocal microscope system, equipped with a HCX PL APO 63× or 100× magnification, numerical aperture 1.40, oil immersion objective, which also allowed the acquisition and analysis of digital images by a dedicated software (Leica AS). When indicated in the figure legends, cell images were reported as the merge of a complete z-stack of the observed field.

### Ca^2+^ imaging

For Ca^2+^ imaging with the cameleon probes, 12 days after plating cells were mounted into an open-topped chamber and maintained under perfusion with a Krebs-Ringer buffer (KRB; in mM: NaCl 125, KCl 5, KH_2_PO_4_ 0.4, MgSO_4_ 1, glucose 5.5, HEPES 20; pH 7.4) containing CaCl_2_ (2 mM), through a temperature-controlled (37 °C) instrument (TC-324B, Warner Instruments,). Cells were stimulated with AMPA (Tocris, 25 μM) for cytosolic and mitochondrial Ca^2+^ measurements, or with 100 μM caffeine for ER Ca^2+^ measurements (both stimuli were carried out in KRB).

Cell were analysed using a DM6000 inverted microscope (Leica) with a 40× oil objective (HCX Plan Apo, NA 1.25). Excitation light produced by a led (LZ1-00UA00-LED, Led Engin) was filtered at the appropriate wavelength (425 nm) through a band-pass filter, and the emitted light was collected through a dichroic mirror (515 DCXR, Chroma Technologie), and a beam-splitter (OES) with emission filters (Chroma Technologies) HQ 480/40 M (for CFP) and HQ 535/30 M (for YFP). The beam-splitter permits the collection of the two emitted wavelengths at the same time, thus preventing any artefact due to uncontrolled movement of the recording chamber and/or intracellular organelles. Images were acquired using an IM 1.4 C cooled CCD (Jenoptik Optical Systems) attached to a 12-bit frame grabber. Synchronization of the excitation source and the CCD was performed through a control unit ran by a custom-made software package (developed by C. Ciubotaru, Venetian Institute of Molecular Medicine, Padova, Italy), which was also used for image acquisition, with an exposure time of 100 ms. The software recorded with-time variations (sampling rate = 1 s^−1^) of the ratio (R) between the acceptor and the donor fluorescence intensity, which was taken as a relative measurement of Ca^2+^ concentration. Peak values of Ca^2+^ transients were reported as the difference between the peak and the baseline R value (ΔR = R_peak _− R_baseline_).

### *In vivo* AAV-mediated delivery of cameleons

For *in vivo* delivery of AAV vectors, we have used P1 mice, in light of previous reports demonstrating much higher transduction rates in newborns than in adult mice^43, 44^. After anaesthesia by topical administration of lidocaine, neonatal hSOD1(WT)-expressing mice (P1) received 50 μl of viral suspension containing 3 × 10^11^ GC/ml of AAV9-Hb9_AB-4mtD3cpv or AAV9-Hb9_AB-D4ER, or vehicle (PBS), into the temporal vein using a Hamilton syringe with a 32-gauge needle. The injections were performed in a Biosafety Level 2 (BL2) laboratory.

Four weeks after the injection, animals were anesthetized by CO_2_ before killing by cervical dislocation, and spinal cords were collected and successively fixed in PFA [24 h, 4 °C, 4% (w/v)]. Fixed tissues were transferred to sucrose [30% (w/v) in PBS, overnight, 4 °C] for cryoprotection. 20 μm-thick sections were serially cut on a cryostat (Leica Microsystems), immunostained for the MN marker SMI32 and counter-stained with the nuclear dye Hoechst 33342, as described previously for immunocytochemical analyses. Finally, slices were mounted in montage solution on a glass coverslip, and observed with a fluorescence stereo-microscope (Leica M205-FA), or with the above described inverted confocal microscope system.

### Statistical analysis

Off-line analysis of FRET data was performed with the ImageJ software. YFP and CFP images were subtracted for the respective background signals, and distinctly analysed after choosing proper regions of interest on selected cells. Subsequently, the ratio between YFP and CFP emission fluorescence intensity (F) was calculated (R = F_535_/F_480_) and reported as %. All the data are representative of at least n (indicated in the figures and/or figure legends) independent experiments, and are reported as mean ± standard error of the mean (SEM). Statistics were performed by unpaired Student’s t test, with a p value < 0.05 being considered statistically significant.

For mouse genotyping, NSC-34 cell culturing, and methods for Western blot analysis and Fura-2-based Ca^2+^ measurements, see Supplementary Information.

## Electronic supplementary material

Supplementary Information
