# Supplementary material for: Generation and validation of novel adeno-associated viral vectors for the analysis of Ca2+ homeostasis in motor neurons
Source: Sci Rep. 2017 Jul 26;7:6521. doi: 10.1038/s41598-017-06919-0 (PMC5529510; doi:10.1038/s41598-017-06919-0)
Supplement: Supplementary file 1 — Supplementary Information [file 41598_2017_6919_MOESM1_ESM.pdf]

Supplementary Information

Generation and validation of novel adeno-associated viral vectors for the analysis of Ca^{2+} homeostasis in motor neurons

Rosa Pia Norante[1#], Maria Lina Massimino[2#], Paolo Lorenzon[1§], Agnese De Mario[1], Caterina Peggion[1], Mattia Vicario[1], Mattia Albiero[3], Maria Catia Sorgato[1,2], Raffaele Lopreiato[1], Alessandro Bertoli[1*]

Supplementary Materials and Methods

Primer sequences.

1) ER-KpnI-F: 5'-TGGCGGTACCatgctgctgcccgtcccccctgctgc
2) ER-HindIII-R: 5'-CGATAAGCTTttacagctcgtccttgccgagagtg
3) AB-MluI-F: 5'-CCGCACGCGTcgctgcgtcgacagatctcatatgg
4) AB-KpnI-R: 5'-TGGCGGTACCgccagtaagcagtggacctgcaggc
5) hSOD1-F: 5'-CATCAG CCCTAATCCATCTGA
6) hSOD1-R: 5'-CGCGACTAACAATCAAAGTGA
7) IL2-F: 5'- CTAGGCCACAGAATTGAAAGATCT
8) IL2-R: 5'- GTAGGTGGAAATTCTAGCATCATCC

Primers (1-4) were used for the cloning of the ER cameleon probe (1-2) or the Hb9_AB promoter (3-4). Restriction sites for KpnI, HindIII and MluI are underlined. Template homologous sequences are reported in lower case. Primers (5-8) were used for mouse genotyping (see below).

AAV titers. AAV9-[Hb9_AB]-D1cpv: 9.53×10^{13} GC/ml; AAV9-[Hb9_AB]-D4ER: 8.83×10^{13} GC/ml; AAV9-[Hb9_AB]-4mtD3cpv: 1.88×10^{13} GC/ml

Mouse genotyping. For the extraction of PCR-ready DNA from mouse embryos, or newborns (p1-2), or fingers samples (from p7 mice), the Mouse Genotyping Kit (KAPA Biosystems) was used, following the manufacturer's instructions. For amplification, 1 µl of extract was diluted in 20 µl of KAPA2G Fast Genotyping Mix containing forward and reverse primers for hSOD1 (hSOD1-F and hSOD1-R, respectively; expected PCR product 236 bp) and interleukin-2 (internal positive control; IL2-F and IL2-R; expected PCR product 324 bp) (0.5 µM each, Sigma, see the above sequences). After a denaturation step (95 °C, 3 min), the PCR mix was subjected to the following cycle: 95 °C, 15 s (denaturation); 61 °C, 30 s (annealing); 72 °C, 45 s (extension); after 35 cycles, PCR was terminated with an incubation at 72 °C (2 min). PCR products were separated by agarose (2% w/v) gel electrophoresis, and amplified bands were visualized by SERVA DNA Stain G.

NSC-34 cells. Immortalised motor neuron-like NSC-34 cells[16], were maintained in a proliferative medium consisting of DMEM supplemented with FBS [(10% (v/v)], glutamine (2 mM), penicillin (100 U/ml), and streptomycin (100 µg/ml). Cells were seeded (10,000 or 4,000 cells for proliferative or

differentiating conditions, respectively) into 13 mm-diameter glass coverslips coated (2 h, 37 °C) with collagen I [Sigma (0.1% (w/v)] in PBS, and grown, depending on the experiment, in proliferation or differentiation medium [the latter containing 1:1 (v:v) DMEM:Ham's F12, supplemented with 1% (v/v) FBS, 1% (v/v) modified Eagle's medium non-essential amino acids (Gibco) and 5 μM retinoic acid]. Cells were transduced with the three different AAV-Hb9_AB-cameleon particles 24 h after plating, and analysed for the expression of cameleons 7 days after transduction.

Western blot. NSC-34 cells, both proliferating of induced to differentiate for different time periods, were homogenized in a buffer containing glycerol [10% (w/v)], sodium dodecyl-sulphate (SDS) [2% (w/v)], Tris/HCl (62.5 mM, pH 6.8), and a protease inhibitor cocktail (Roche). After determining the total protein concentration by a bicinchoninic acid assay (Pierce), lysates were adjusted to an equal protein concentration using reducing (dithiothreitol, 50 mM) Laemmli sample buffer. After boiling (5 min), proteins were separated by SDS polyacrylamide-gel-electrophoresis [12% (w/v) acrylamide-N,N'-methylenebisacrylamide (37.5:1)], and electro-blotted onto nitrocellulose membranes (0.45 μm pore size, Biorad). To verify equal loading and transfer, nitrocellulose membranes were stained with Ponceau red, and, after de-staining, were incubated (1 h, RT) with blocking solution [Tris-buffered saline added with 0.1% (w/v) Tween-20 (TBS-T) containing 5% (w/v) non-fat dry milk]. Subsequently, membranes were probed (overnight, 4 °C) with a rabbit mAb to the MN marker choline acetyl-transferase [1:1000 in TBS-T added with 3% (w/v) BSA; Abcam, cat. n. EPR13024(B)]. After 3 washings (10 min each in TBS-T), membranes were incubated (1 h, RT) with a horseradish peroxidase-conjugated anti-rabbit secondary antibody [Santa Cruz Biotechnology, cat. n. sc-2004; 1:3000 in TBS-T added with BSA 1% (w/v)]. Immunoreactive bands were visualized and digitalized by means of a digital Kodak Image Station, using an enhanced chemiluminescence reagent kit (Millipore).

Fura-2-based Ca^{2+} imaging. For cytosolic Ca^{2+} measurements with the chemical probe Fura-2, primary spinal cord cells (on day 2 after plating) were transfected (using the Lipofectamine-2000 reagent) with the Bg.Hb9_AB_GFP plasmid[29] driving the expression of the green fluorescent protein (GFP) specifically in MNs. After 12 days from plating, cells were incubated (40 min, 37 °C) with Fura-2/AM (1 μM, Life Technologies), pluronic F-127 (0.02%, Molecular Probes), and sulfinpyrazone (200 μM, Sigma) in a modified Krebs-Ringer buffer (mKRB; in mM: NaCl 140, KCl 2.8, $MgCl_2$ 1, $CaCl_2$ 2, 4-[2-hydroxyethyl]-1-piperazineethanesulfonic acid 10, glucose 11; pH 7.4), and then further incubated in mKRB (20 min, RT). Fura-2-loaded, GFP-positive, MNs were then processed for Ca^{2+} imaging by an ultraviolet-permeable objective (40× magnification, Olympus Biosystems GmbH, Planegg, Germany) on

an inverted fluorescence microscope (Zeiss Axiovert 100, Jena, Germany). Alternating excitation wavelengths of 340 and 380 nm were applied by a monochromator controlled by the *TILLvision* software (TILL Photonics, Martinsried, Germany). A neutral density filter (UVND 0.6, Chroma, USA) was used in the excitation pathway, and the emitted fluorescence was measured at 510 nm. Images were acquired every 5 seconds, with 200 ms exposure time at each wavelength, by a *TILL-Imago* camera controlled by a dedicated software. Cells, bathed in mKRB throughout the recordings, were stimulated with AMPA (25 μM) by means of a gravity-controlled perfusion system. The ratio of the emitted fluorescence intensities (F340/F380), calculated by the acquisition software, was averaged offline.

Supplementary Figure S1

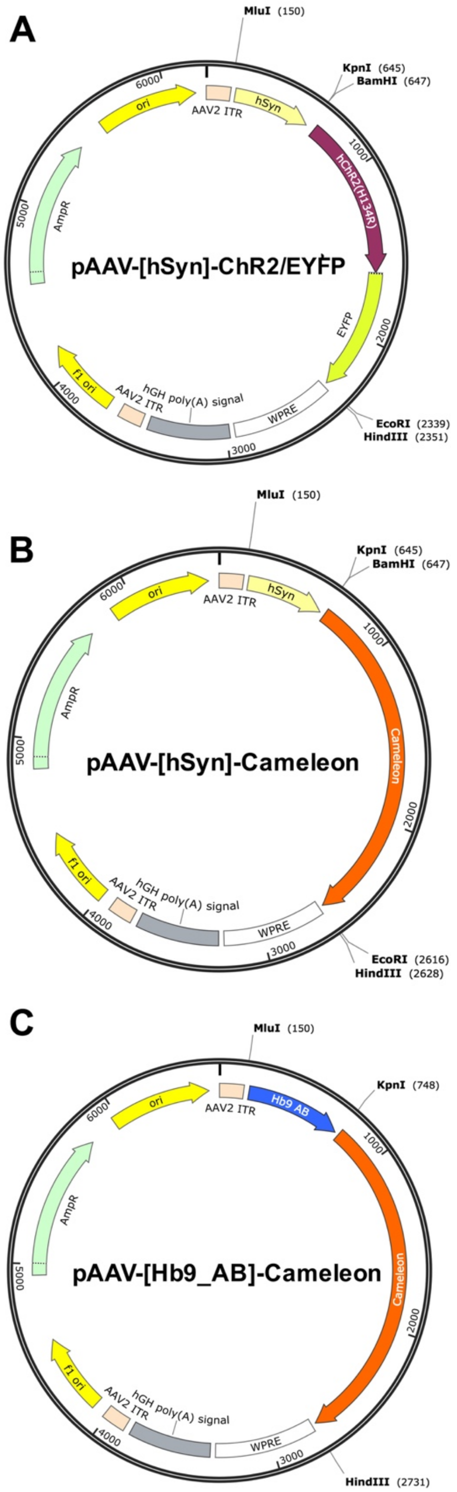

Figure S1. Maps of the pAAV plasmids. The adeno-associated virus plasmids (pAAV) used in this work have been generated by a two-step cloning strategy. We firstly inserted the cameleon coding sequence at the BamHI-EcoRI (for the cytosolic probe, D1cpv) or KpnI-HindIII for the ER lumen probe, D4ER) sites of the pAAV-[hSyn]-ChR2/EYFP vector (A), thus producing the pAAV-[hSyn]-Cameleon plasmids (B), able to express the Ca^{2+} probes under the control of the pan-neuronal human synapsin1 (hSyn) promoter. Then, we substituted the [hSyn] sequence in the above plasmids, or in the already available pAAV-[hSyn]-4mtD3cpv (coding for the cameleon targeted to the mitochondrial matrix, 4mtD3cpv), with a minimal MN-specific promoter derived from the homeobox Hb9 gene promoter (Hb9_AB; Peviani et al., 2012, J. Neurosci. Methods 205, 139-147, doi: 10.1016/j.jneumeth.2011.12.024), by using the MluI-KpnI sites, finally generating the pAAV-[Hb9_AB]-Cameleon plasmids (C), either expressing the cytosolic, ER or mitochondrial probe (consisting of 1962, 1986, and 2373 bp, respectively).

Supplementary Figure S2

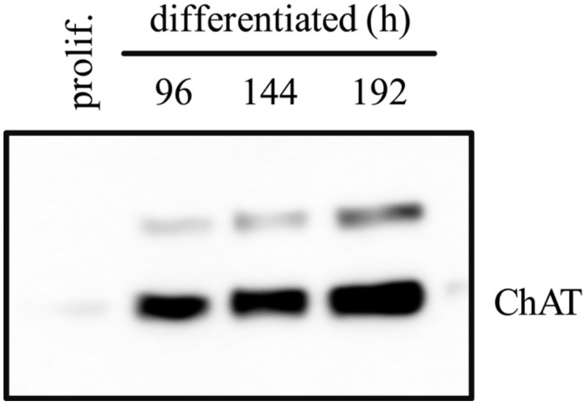

Figure S2. *In vitro* differentiation of NSC-34 cells. NSC-34 cells were cultured under proliferating (prolif.) or differentiating (by growth in the presence of retinoic acid 5 μM for the indicated period of time) conditions. Cell lysates were then subjected to Western blot analysis for the expression of the MN marker choline acetyl-transferase (ChAT). After 96 h of retinoic acid treatment cells already express a substantial quantity of ChAT, which further increases at 144 and 192 h (the time at which cells were analysed for the expression of cameleons), and is instead barely detectable in proliferating cells (i.e., not exposed to retinoic acid).

Supplementary Figure S3

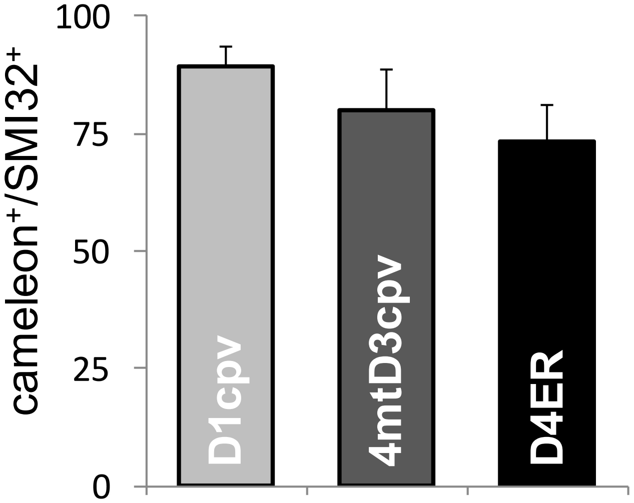

Figure S3. The cameleon probes are efficiently expressed in motor neurons in mouse spinal cord primary cultures. Primary cell cultures from the mouse spinal cord were transduced with the AAV vectors coding for the cameleons targeted to the cytosol (D1cpv), the mitochondrial matrix (4mtD3cpv) or the ER lumen (D4ER). After 12 days of culturing, cells were immunostained for the motor neuron (MN) marker SMI32 and counter-stained with the nuclear fluoro-probe Hoechst 33342, as described in Figure 3. Cell images – acquired by confocal microscopy – were then quantitatively analysed for the presence of the cameleon probes and the expression of SMI32. The bar diagram, reporting the ratio between cameleon-expressing, SMI32-positive, cells (cameleon^{+}) over the total SMI32-positive cells (SMI32^{+}), indicates that more than 70% of SMI32-expressing MNs are transduced with each Ca^{2+} probe. Data are reported as mean ± standard error of the mean (SEM); n ≥ 9 [accounting for 3 technical replicates × at least 3 biological replicates (i.e., independent spinal cord cultures)] for each cameleon probe. Almost identical results were obtained by averaging the different technical replicates for each biological replicate, and then calculating the mean of the biological replicates for each cameleon construct.

Supplementary Figure S4

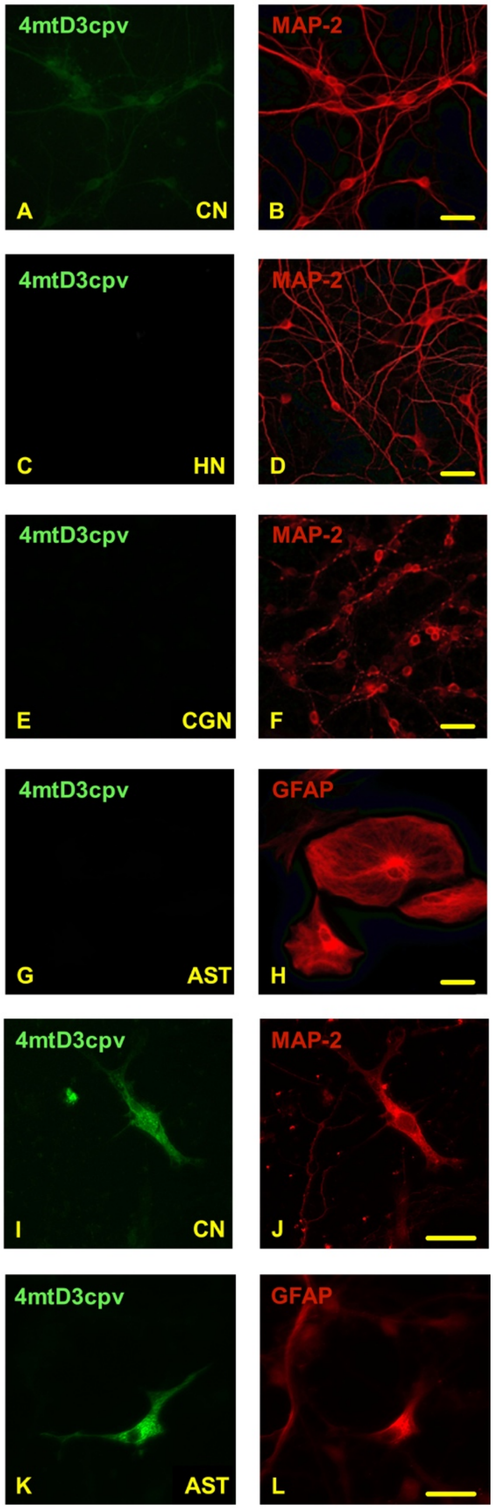

Figure S4. The cameleon probes driven by the MN-specific promoter are not expressed in other neuronal types, nor in astrocytes. Primary murine cell cultures of cortical (CN, panels A,B,I,J) or hippocampal neurons (HN, panels C,D), cerebellar granule neurons (CGN, panels E,F), or spinal astrocytes (AST, panels G,H,K,L) were transduced with the MN-specific promoter-containing AAV vectors (panels A-H), or plasmidic vectors containing an ubiquitous [cytomegalovirus (CMV), panels I-L] promoter, coding for the cameleon targeted to the mitochondrial matrix. After 7 days (CN and HN) or 4 days (CGN) of culturing, or 4 days after re-plating (AST), cells were fixed, permeabilised, and immunostained with antibodies to the neuronal marker MAP-2 or the astrocytic marker GFAP.

Images of cells were then collected by a fluorescence microscope after excitation at either λ = 488 nm for visualising the fluorescent Ca^{2+} probes (green signal, panels A,C,E,G,I,K), or λ = 543 nm for visualising MAP-2- or GFAP-positive cells (red signal, panels B,D,F,H,J,L). The absence of green signal (in panels A,C,E,G) indicates that the MN-specific promoter does not drive the expression of the cameleon probe in these cell types. The expression of the probe, however, can be accomplished in both CN and AST by using the CMV promoter (panels I-L). A higher yield of expression was obtained in CN by use of the AAV vector containing the pan-neuronal human synapsin1 promoter (data not shown). Scale bar = 20 μm (in A-J); 40 μm (in K,L).

Supplementary Figure S5

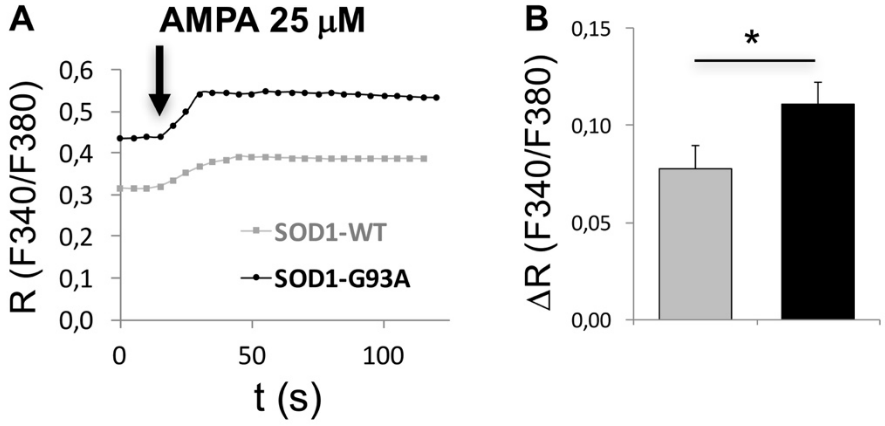

Figure S5. Fura-2-based Ca^{2+} measurements confirm the higher cytosolic Ca^{2+} transients induced by AMPA in primary spinal cord MNs from ALS mice than in the healthy counterpart. Primary spinal cord cultures from hSOD1(WT) or hSOD1(G93A) mice were transfected (on day 2 after plating) with the transfected with the Bg.Hb9_AB_GFP plasmid. After 12 days of culturing, Fura-2-based measurements were performed, after cell loading with the chemical Ca^{2+} dye, on (GFP-positive) MNs. The ratio (R) between the fluorescence intensities at the two alternating excitation wavelengths (F340/F380), acquired by means of a suitable computer-assisted fluorescence microscope, was calculated by the data acquisition software, allowing the comparison of cytosolic Ca^{2+} movements following stimulation with AMPA (25 μM, in the presence of 2 mM $CaCl_2$) at the time-point indicated by the arrow, between hSOD1(WT) or hSOD1(G93A) MNs. Panel A reports the average traces of the Ca^{2+} responses to the stimulus (for the sake of clarity, error bars are not reported), while the bar diagram of panel B reports the mean difference between F340/F380 ratios ($\Delta R = R_{peak} - R_{baseline}$) following the stimulus. Fura-2-based Ca^{2+} measurements corroborate the cameleon-based finding (see Fig. 4) that hSOD1(G93A)-expressing MNs have a significantly higher cytosolic Ca^{2+} response following AMPA stimulation compared to the hSOD1(WT) counterpart. Data are reported as mean ± SEM; n =10 (for SOD1-WT), 6 (for SOD1-G93A), from at least 3 independent cultures for each genotype; * $p < 0.05$, Student's t-test.
